# Two cases of meningocele and meningoencephalocele in Jeju native pigs

**DOI:** 10.1186/s12917-015-0404-y

**Published:** 2015-04-09

**Authors:** In-Cheol Cho, Yong-Sang Park, Jae-Gyu Yoo, Sang-Hyun Han, Sang-Rae Cho, Hee-Bok Park, Kyong-Leek Jeon, Kyoung-Ha Moon, Han-Seong Cho, Tae-Young Kang

**Affiliations:** National Institute of Animal Science, Rural Development Administration, Jeju, 690-150 Republic of Korea; College of Veterinary Medicine, Jeju National University, Jeju, 690-756 Republic of Korea; Educational Science Research Institute, Jeju National University, Jeju, 690-756 Republic of Korea; Division of Animal and Dairy Science (Brain Korea 21 plus Program), Chungnam National University, Daejeon, 305-764 Republic of Korea

## Abstract

**Background:**

Meningocele and meningoencephalocele of the skull are congenital deformities. Various species, such as pigs, dogs, and cats, are susceptible to congenital meningocele and meningoencephalocele and the incidence is higher in large white and landrace pigs.

**Case presentation:**

In this study, swelling was observed in the fontanel areas of the median planes of the skull cap in two female piglets of the same litter. Gross clinical examination, neurological examination, computed tomography (CT), and magnetic resonance imaging (MRI) were conducted on the symptomatic piglets. The gross clinical and neurological examinations revealed no specific findings, except for the swellings. According to the CT results, the length of the defect on the sagittal section of the skull was 4.7 mm in case 1 and 20.62 mm in case 2. Connected flow between the skull swellings and the cerebrospinal fluid (CSF) of the lateral ventricles was observed, and partial herniation was identified in case 2. On MRI, CSF with high T2 signals was identified in the arachnoid spaces between the cerebrum and the cerebellum in the two cases, which is consistent with intracranial hypertension. The size of the swelling formed in the parietal bones was 1.6 × 1.1 × 1.8 cm^3^ (case 1) and 1.2 × 1.38 × 1.7 cm^3^ (case 2). The increase in intracranial pressure was more obvious in case 2 than in case 1, and was accompanied by posterior displacements of the mesencephalon and cerebellum.

**Conclusions:**

Case 1 was diagnosed as meningocele resulting from meningeal herniation and case 2 was diagnosed as meningoencephalocele caused by brain tissue herniation.

## Background

Meningocele and meningoencephalocele of the skull are congenital deformities. These deformities, which are observed as cyst-like swellings in the median part of the skull cap, occur very rarely. The intracranial material protrudes through a spontaneous cavity, such as the anterior fontanelle [1], and they are classified as encephalocele, meningocele, or meningoencephalocele according to the cranial bifida [2]. The condition is called meningocele when only cerebrospinal fluid (CSF) exists in the meningeal swelling, and it is called meningoencephalocele when brain tissues coexist in the swelling [3]. Dysraphism or a defect of the anterior fontanelle area triggers meningoencephalocele and meningocele. These defects take place in relation to agenesis of the surface ectoderm and neuroectoderm [2,4,5]. Congenital meningocele and meningoencephalocele occur in various animals, including horses, pigs, dogs, cats, and goats [1,4-10]. Their incidence is higher in large white and landrace pigs [1,5,11,12]. Experimentally, the incidence of meningoencephalocele upon crossing with a group with meningoencephalocele is between 0.95% and 1.37% [2,3]. Encephalocele and meningocele mostly occur in the suture line of the frontal region, and sometimes in the occipital region and the posterior occipital crest, whereas meningoencephalocele largely occurs in the occipital region [2,13]. Meningocele and meningoencephalocele have been diagnosed in humans and various animals by using computed tomography (CT) and magnetic resonance imaging (MRI). The occurrence of meningocele and meningoencephalocele in the indigenous pigs of Jeju Island has not been previously reported. In this study, we examined the brains of two Jeju Island indigenous pigs by using CT and MRI to diagnose meningocele and meningoencephalocele.

### Materials and methods

We obtained two female piglets (age, 2 days; weight, ~1.4 kg) from a single Jeju native pig litter from a farm in Korea. The physical examination revealed a sac-like protrusion with fluctuant swelling in the frontoparietal region of the skull vault and a palpable skull defect on the top of the piglets’ heads. A vault swelling may protrude through a normal opening, such as the anterior fontanelle. An ultrasound scanner (ProSoundAlpha 6, Hitachi Aloka, Japan) was used to examine the fluctuant swelling. The swelling appeared to be filled with anechoic fluid, which was likely CSF. The presence of brain tissue in the swelling could not be confirmed by the use of ultrasound examination and radiograph. Therefore, it was difficult to differentiate between meningocele and meningoencephalocele by using these methods. CT imaging (Asteion Super 4, Toshiba, Japan) and MRI (Vet-MR, Esaote, Italy) were performed to evaluate the swelling in the skull while the animals were under sedation with azaperone (Stresnil, Janssen Pharmaceutica, Belgium). The Animal Care Committee at the National Institute of Animal Science approved all experimental procedures (Approved No. 2014-095).

## Case presentation

**Figure 1:**
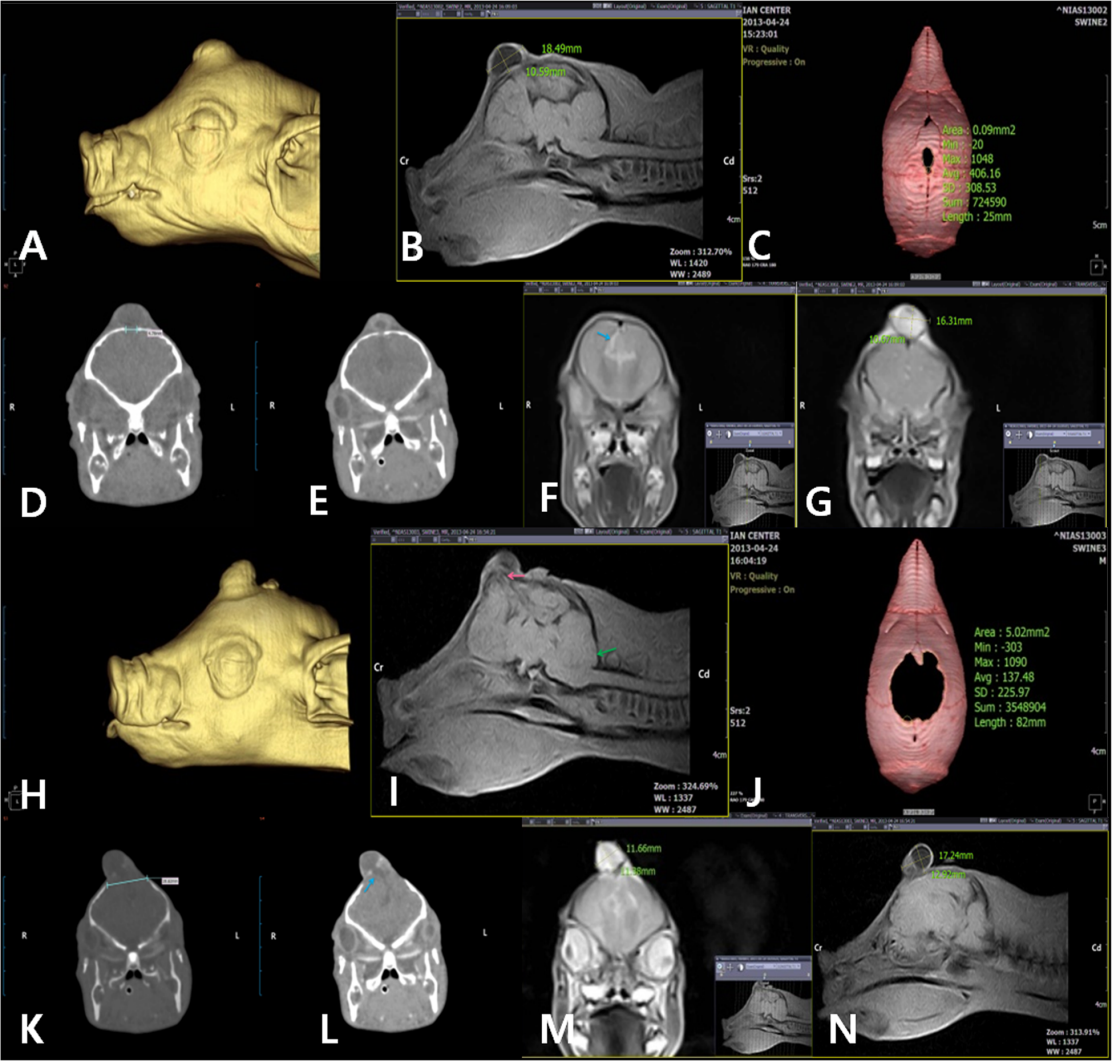
**CT and MRI findings of the pig skull in case 1 (A–G) and case 2 (H–N).**Three-dimensional volume reconstruction is shown in **A** (case 1) and **H** (case 2). The MRI of the pig’s skull is shown in **B**, **F**, **G**, **I**, **M**, **L** and **N**. The imaging of the pig skull is shown in **C**, **D**, **E**, **K**, **L**, and **M**. **B**, The size of the swelling on the median sagittal plane on MRI was 18.49 × 10.59 (L × H, mm). **C** and **J**, Imaging of three-dimensional volume reconstruction of the pig skull defect, specifically a canal with irregular edges. **D**, Transverse-plane section through the skull showing the bone defect in case 1. The length of the defect was 4.78 mm, and a cyst containing matter of fluid density was apparent. **E** and **F**, The matter in the swelling was related to the cerebral ventricle. **G**, The size of the swelling on the cross-section was 10.67 × 16.31 (L × H, mm). **I** and **L**, Transverse-plane section through the skull showing the bone defect in case 2. The herniation of the brain tissues from the median sagittal plane to the skull defect was apparent. Posterior displacement of the cerebellum due to increased intracranial pressure was evident. The size of the swelling was 17.24 × 12.92 (L × H, mm). The transverse cross-section of case 2. The length of the defect was 20.62 mm. A swelling with matter of liquid density was apparent. Transverse cross-section of case 2 on MRI. A swelling with T2 high/T1 low signals was apparent. The size of the protruding swelling was 11.66 × 11.38 (L × H, mm).

Case 1: In a 2-day old female piglet weighing approximately 1.4 kg, lesions connected with the swelling in the brain parenchyma of the prefrontal level were observed on MRI. The subarachnoid space between the cerebrum and cerebellum was filled with fluid, and the CSF was open and flowing. In addition, the corpus callosum was open, and the CSF flow measured by the MRI showed that the space was connected to the lateral ventricle (Figure 1D,E,F). On CT, the fissure lines of the skull and bone discontinuity were disappeared, and the regions in which the fissure lines met was identified (Figure 1C,D). A swelling containing matter of liquid density at the dorsolateral parietal bone was identified, and it was connected to the cerebral ventricle. The length of the defect in the cross-section of the skull was measured at 4.78 mm (Figure 1D). On MRI, a swelling with high T2/low T1 signals was identified in the dorsolateral parietal bone in the transverse cross-section. The size of the swelling was 10.67 × 16.31 (L × H, mm) (Figure 1G). Furthermore, the CSF flow of the lateral ventricle was connected with the defect of the skull. A swelling with high T2/low T1 signals was identified on the dorsolateral parietal bone from the median sagittal plane (Figure 1E,F), and bone discontinuity disappearance of the skull was also observed. The swelling on the median sagittal plane was 18.49 × 10.59 (L × H, mm). With CSF leakage through the defective parietal bone, the mass observed in the parietal region formed a swelling with high T2/low T1 signals on MRI. The size of the swelling was 1.6 × 1.1 × 1.8 (L × H × W, cm). Case 1 was diagnosed as meningocele by meningeal herniation based on crania bifida of the skull in the anterior fontanelle region of the parietal bone. Morphologically, the case was diagnosed as extracranial (transcalvarial) herniation.

Case 2: Case 2, which was from the same litter as case 1, had two swellings in the frontal lobe region. As in case 1, both MRI and CT were used to examine case 2. The fluid of the case 1 swelling was an ahemorrhagic-serous exudate, but the fluid in case 2 was a hemorrhagic exudate. We observed characteristic changes in the brain structure, such as findings of ventrical herniation resulting from the exchange of CSF and posterior displacement of the cerebellum region due to increased brain pressure in the prosencephalic cavity. The length of the defective area of the transverse cross-section on CT of the skull was 20.62 mm (Figure 1K). The size of the protruding swelling was 12.02 × 10.62 (L × H, mm), and the matter of the swelling was connected with the cerebral ventricle.

On MRI, a swelling with high T2/low T1 signals in the transverse cross-section was identified, and the size of the protruding cyst was 11.66 × 11.38 (L × H, mm) (Figure 1M). Herniation of the brain tissue from the median sagittal plane to the defective area of the skull and posterior displacement of the cerebellum resulting from increased intracranial pressure were observed (Figure 1I). The size of the swelling on the median sagittal plane was measured at 17.24 × 12.92 (L × H, mm) (Figure 1L). As in case 1, case 2 had crania bifida of the skull in the anterior fontanelle region of the parietal bone. However, case 2 was diagnosed as meningoencephalocele because it was accompanied by herniation of the brain and the meninges encephali due to increased intracranial pressure. Morphologically, this case was diagnosed as extracranial (transcalvarial) herniation.

### Discussion

Encephalocele is a deformity resulting from the herniation of brain tissue through a skull defect. Encephalocele and meningoencephalocele reflect imperfect osteogenesis of the skull and largely accompany protrusions of the brain structure [2,13]. The classification of encephalocele depends on the degree of brain tissue herniation. The defect is classified as cranium bifidum when only a skull defect is present. Herniation of the cranial dura mater through the defect is classified as meningocele, and herniation of the cranial dura mater and brain parenchyma are classified as meningoencephalocele. In humans, cranium bifidum occurs in one to four out of 10,000 live births [13]. Meningoencephalocele that caused the herniation of the cerebral tissue and meninges was found to occur in the occipital region, the frontal region, and the temporal region in 75%, 12%, and 13% of cases, respectively. Rare sites for protrusions are through the base of the skull, orbits, nose, or mouth [13]. Meningocele and meningoencephalocele are the result of a focal failure of the neuroectoderm and surface ectoderm to separate during fetal development, and these deformities have many potential causes, including genetic factors, nutritional deficiencies, and exposure to teratogenic agents during gestation [1,14,15]. Hereditary meningocele and meningoencephalocele cases have been reported in pigs and cats, and both of these hereditary diseases showed incomplete penetrance [3,5]. Cranium bifidum can be diagnosed by radiography, but differentiating between meningoencephalocele and meningocele with radiography and ultrasonography is difficult. However, meningoencephalocele and meningoceleare easily recognized by CT. CT scans enable differentiate between a meningocele and meningoencephalocele and measurement of the diameter of the defect. CT scans are a simple and valuable non-invasive diagnostic technique in animals. Nonetheless, when describing structural changes of the brain, CT scans should be combined with findings of changes in the brain parenchyma via MRI. An increase in intracranial pressure is commonly discovered in CT and MRI findings. CSF leakage is considered to occur in relation to the neural canal defect, and such neural canal defects are also associated with the size of the skull cap defect into meningoencephalocele and meningocele.

## Conclusion

In this study, CT and MRI were used to make a diagnosis of encephalocele cases in pigs. The length of the defect on the sagittal section of two piglets’ skull was estimated by using CT. Case 1 had a defect 4.7 mm in length and connected flow between the skull swellings and CSF. Case 2 had longer defect length (20.62 mm) than case 1 and partial herniation. On MRI imaging, both cases showed high T2 signals of CSF in the arachnoid spaces between the cerebrum and the cerebellum. The increase in intracranial pressure was more obvious in case 2 than in case 1, and accompanied posterior displacements of the mesencephalon and cerebellum. Case 1 was diagnosed as meningocele resulting from meningeal herniation, and case 2 was diagnosed as meningoencephalocele caused by brain tissue herniation.
